# Precision intervention for sarcopenia

**DOI:** 10.1093/pcmedi/pbac013

**Published:** 2022-05-10

**Authors:** Xiaolei Liu, Jirong Yue

**Affiliations:** National Clinical Research Center for Geriatrics, West China Hospital, Sichuan University, Chengdu 610041, China; National Clinical Research Center for Geriatrics, West China Hospital, Sichuan University, Chengdu 610041, China

Dear Editor,

Sarcopenia is an aging-related disease characterized by progressive muscle mass loss, decreasing muscle strength, and physiological muscle function decline. It is associated with multiple adverse outcomes, including falls, fractures, physical disability, and death. The new code in ICD-10-CM (M62.84) in 2016 signifies its being recognized as a disease and drawing attention to the condition in this ever-aging society. The prevalence of sarcopenia in the elderly is ∼6.8%–25.7% for Asia^1^ and, in particular, 8.9%–38.8% for China.^2^ The mechanism of sarcopenia is complex and includes hormonal changes, nutritional deficiencies, chronic inflammation, neuromuscular function decline, and decreased physical activity. While no specific drugs have been approved to treat sarcopenia, ten pharmacological interventions have been identified to ameliorate the condition in the elderly, including growth hormone, growth hormone-releasing hormone, vitamin D, dehydroepiandrosterone, combined estrogen–progesterone, testosterone-growth hormone, pioglitazone, testosterone, insulin-like growth factor-1, and angiotensin-converting enzyme inhibitors.^3^ Possible drugs for sarcopenia are under development (Table 1).^4^ As a result, understanding the mechanisms of sarcopenia is critical for drug development.

**Table 1.** Current development of drugs for sarcopenia.^4^

Drug name	Target	Company name	Current status
Bimagrumab (antibody)	Activin receptor type 2B	Novartis AG	Leads to significant reductions in fat mass, increases in lean body mass and metabolic improvements over 48 weeks in overweight or obese patients with type 2 diabetes. (February 2017 to May 2019, 48-week, phase 2 randomized clinical trial).
Trevogrumab (antibody)	Myostatin	Regeneron Pharmaceuticals Inc.	Leads to significant change in total lean body mass (phase 2).
Sarconeos (natural active ingredients)	Proto-oncogene protein c-MAS-1, MAS receptor	Biophytis SAS	Leads to better muscle function in animal models of muscular dystrophies with good tolerability profile (phase 1).
ARM-210 (small molecule)	Ryanodine receptor	ARMGO Pharma Inc.	Treats Becker and limb-girdle muscular dystrophies and cachexia.
NA (cell therapy)	Enzyme/protein replacement therapy	Immusoft Corporation	Immune system programming technology.
NT-1654 (a C-terminal fragment of mouse agrin)	The agrin/Lrp4/MuSK system	Neurotune AG	Leads to accelerating muscle re-innervation after nerve crush.
AVGN7 (gene therapy)	Activin receptors	AAVogen Inc.	Contains SMAD7 gene that could stop gene expression for muscle wasting.
ATA 842 (antibody)	Myostatin, activin	Amgen Inc.	Leads to increased muscle mass and muscle strength in mouse model after 4 weeks.
VB-102 (protein)	NA	Vibe Pharmaceuticals LLC	Has the potential to regenerate muscle and bones.
Peptide of follistatin	Furin, Janus kinase 3, myostatin	MYOS RENS Technology Inc.	A myostatin inhibitor for the treatment of sarcopenia.
Monovalent FSTL3-Fc fusion protein (mono-FSTL3-Fc)	Growth factor-β family ligands	NA	Leads to an increase of systemic muscle mass in mice using intraperitoneal administration.
AAV gene therapy	Myostatin	BioViva	Has potential ability in the modulation of myostatin expression.
TEI-SARM2	Androgen receptor	Teijin Pharma Ltd.	A selective androgen receptor modulato.

NA, not applicable.

The treatment of sarcopenia currently focuses on nutrition and exercise interventions. However, the clinical evidence is very limited and many questions still remain unanswered. For example, how can the safety and compliance of exercise interventions be ensured according to stress adaptability? Besides, a large percentage of sarcopenic patients cannot live up to recommended degrees of both nutritional food intake and physical activity, resulting in numerous problems. Therefore, for elderly patients with sarcopenia with different conditions, individualized intervention and management strategies are urgently needed according to the patient's metabolic and digestive functions. Food components with anti-inflammatory properties, such as probiotics and traditional Chinese medicine prescriptions, should be considered for intervention.

Sarcopenia is associated with different genotypes. For example, in sarcopenia patients, the X allele of the alpha-actinin-3 (ACTN3) genotype was found to be more associated with decreased thigh muscle volume compared with the RR allele of the ACTN3 genotype.^5^ In addition, angiotensin I-converting enzyme (ACE) gene insertion/deletion (I/D) polymorphism has been associated with improvements in performance and exercise duration in a variety of populations. Specifically, the I allele of ACE genotype is associated with endurance-orientated events, while the D allele is associated with strength- and power-orientated performance.^6^ Another gene associated with sarcopenia is vitamin D receptor (VDR), and FF carriers have double the risk of having sarcopenia compared with carriers of the f allele.^7^ Other genetic variations associated with sarcopenia include the tumor necrosis factor-α (TNFα), insulin-like growth factor-1 (IGF1), insulin-like growth factor binding protein 3 (IGFBP3), uncoupling protein-2/3 (UCP2/3), apolipoprotein E (APOE), and ciliary neurotrophic factor/R (CNTF/R) genes.^8^ Thus, determining the underpinning skeletal muscle genotype is important in precision treatment/intervention for sarcopenia.

Today's new technologies, including smartphone software and wearable devices, neuromuscular electrical stimulation, smart house, 3D printed foods, and interactive and virtual reality (VR) games, help to make individualized sarcopenia management possible.^9^ These techniques can help older adults with sarcopenia remain independent and get adequate physical activity and nutrition depending on individualized requirement. For example, smartphone software and wearables can track activity metrics including steps, distance, and intensity of physical activity, helping clinicians to obtain activity data remotely and monitor patient compliance and exercise progress. Other technologies, such as whole-body vibration training (WBVT) and neuromuscular electrical stimulation (NMES), can help improve muscle strength. In addition, robotic devices also facilitate passive or active training for sarcopenia patients.^10^

Furthermore, a smart home includes many connected devices that can help the elderly achieve independence. For example, smart refrigerators have the function to help older adults maintain adequate nutrition by monitoring daily dietary intake, providing older adults with personalized meal plans, and buying food through online systems. In addition, 3D food printers are emerging as a new way to provide personalized nutrition to older adults. Meals may be printed at home and customized to provide nutrient contents that can help older adults meet dietary prescriptions. Besides, VR and interactive video games can supply a new platform for exercise programs, providing more enjoyable experiences than a typical exercise regime in treating sarcopenia. Of course, further research is needed to determine the role of currently available technologies in managing sarcopenia.

Precision medicine is defined as a novel medical paradigm focusing on personalized, predictive, preventive, and participatory approaches, depicting a brand new way to treat sarcopenia. A person's genotype and other characteristics will determine how to individualize the management of sarcopenia. Precision medicine will improve the life quality of a large population of sarcopenia patients.

## Conflict of interest

None declared.
